# Chromogenic In Situ Hybridization and p16/Ki67 Dual Staining on Formalin-Fixed Paraffin-Embedded Cervical Specimens: Correlation with HPV-DNA Test, E6/E7 mRNA Test, and Potential Clinical Applications

**DOI:** 10.1155/2013/453606

**Published:** 2013-11-24

**Authors:** Roberta Zappacosta, Antonella Colasante, Patrizia Viola, Tommaso D'Antuono, Giuseppe Lattanzio, Serena Capanna, Daniela Maria Pia Gatta, Sandra Rosini

**Affiliations:** ^1^Cytopathology Unit, Experimental and Clinical Sciences Department, “G. d'Annunzio” University of Chieti-Pescara, Via dei Vestini, 66100 Chieti, Italy; ^2^Surgical Pathology Unit, “SS Annunziata” Hospital, ASL2 Abruzzo, Chieti, Italy

## Abstract

Although HPV-DNA test and E6/E7 mRNA analyses remain the current standard for the confirmation of human papillomavirus (HPV) infections in cytological specimens, no universally adopted techniques exist for the detection of HPV in formalin-fixed paraffin-embedded samples. Particularly, in routine laboratories, molecular assays are still time-consuming and would require a high level of expertise. In this study, we investigated the possible use of a novel HPV tyramide-based chromogenic in situ hybridization (CISH) technology to locate HPV on tissue specimens. Then, we evaluate the potential usefulness of p16^INK4a^/Ki-67 double stain on histological samples, to identify cervical cells expressing HPV E6/E7 oncogenes. In our series, CISH showed a clear signal in 95.2% of the specimens and reached a sensitivity of 86.5%. CISH positivity always matched with HPV-DNA positivity, while 100% of cases with punctated signal joined with cervical intraepithelial neoplasia grade 2 or worse (CIN2+). p16/Ki67 immunohistochemistry gave an interpretable result in 100% of the cases. The use of dual stain significantly increased the agreement between pathologists, which reached 100%. Concordance between dual stain and E6/E7 mRNA test was 89%. In our series, both CISH and p16^INK4a^/Ki67 dual stain demonstrated high grade of performances. In particular, CISH would help to distinguish episomal from integrated HPV, in order to allow conclusions regarding the prognosis of the lesion, while p16^INK4a^/Ki67 dual stain approach would confer a high level of standardization to the diagnostic procedure.

## 1. Introduction

HPV infection is recognised as the necessary cause of cervical intraepithelial lesions (CIN) and invasive squamous cell carcinoma (SSC). However, only a minority of viral infections ever results in neoplastic lesions. It is well known that the majority of HPV infections may be cleared by the immune system, and that certain high-risk (HR) HPV types (HPV 16, 18, 31, 33, 45, and 54) are significantly more common among high-grade lesions and carcinomas [1].

The most important factor in CIN progression is certainly the integration of HPV sequences into the host genome with the loss of E2 tumor suppressor gene. E2 physiologically regulates the expression of E6 and E7 oncogenes. There is consensus that integration is common in high-grade CIN and cancer, while it is infrequent or is lacking in low-grade CIN. HPV integration, disrupting cell-cycle control and escaping immune system surveillance, would induce stochastic accumulation of genetic aberrations, leading to CIN progression.

Recently, a wide range of molecular techniques has been evaluated on cytological specimens, to improve cervical cancer screening strategies [2, 3]. HPV-DNA test showed a high sensitivity in identifying CIN, but it still lacks clinical specificity, due to the high prevalence of transient infection [2]. E6/E7 mRNA test, targeting patients at higher risk of CIN progression, demonstrated to be more specific than DNA test in stratifying the risk for cancer development [4]. 

On tissue specimens, the ideal test for the detection of HPV has not been established yet, although different assays have been analyzed (i.e., PCR, in situ hybridization, ISH). Potential useful marker should target viral genome or related proteins (i.e., DNA, mRNA) or should identify host cell's products whose expression would be stimulated by HPV infection. In this context, immunohistochemical (IHC) localization of p16^INK4a^ (henceforth p16) seems to represent one of the most widely investigated tool. 

p16 is a tumor suppressor protein playing a crucial role in cell-cycle regulation. p16 prevents the phosphorylation of the retinoblastoma protein (pRb) by inhibiting cyclin-dependent kinases CDK4 and CDK6. Physiologically, non-phosphorylated pRb binds the transcription factor E2F, thereby preventing E2F stimulation of cell progression into S phase. The functional inactivation of pRb by HPV-E7 oncoprotein induces E2F factor release that becomes subsequently free to drive cell-cycle progression towards S phase.

All the above mentioned markers and technologies are a matter of controversy, each having their advantages and drawbacks. 

PCR is considered the most effective method for HPV-DNA detection, but some problems still exist in routinely practice: DNA extraction compromises the preservation of tissue architecture [5]; moreover, it requires a high of expertise and strict laboratory conditions, to avoid contaminations [6]. ISH is cheap and relatively easy to perform. It would permit the detection of HPV-DNA, as well as the preservation of histological pattern. On the other hand, ISH lacks in sensitivity (limit of 10–50 DNA copy/cell) [7, 8]. To by-pass this problem, a tyramide-based signal amplification kit, based on HPV chromogenic in situ (CISH) technology, has been developed [5].

p16 demonstrated to be useful as surrogate biomarker of HPV integration and E7 overexpression. However, pitfalls such as positive staining by nondysplastic cells would limit its clinical accuracy. Recently, a novel concept of biomarker based on the combination of p16 and Ki-67 detection in cervical cytology specimens (p16/Ki-67 double stain) has been proposed. Under physiological conditions, the coexpression of these proteins does not occur, since they typically induce opposite effects [6]. Simultaneous expression of both markers within the same cervical cell would indicate HPV-dependent deregulated cell cycle. Only limited results are available for p16/Ki67 assay [6, 9, 10]; all of these concerning its potential utility on cytological samples. To our knowledge, there are no data regarding the feasibility of p16/Ki67 double stain on histological specimens.

Basing on this background, in the first phase of this study, we aimed to analyze analytical and diagnostic accuracies of the novel CISH technology in detecting viral DNA and in identifying HPV physical status on formalin-fixed and paraffin-embedded tissue. To do that, CISH results were compared with results obtained from HPV-DNA test and HPV-mRNA test. 

In the second phase, we assessed the potential usefulness of CINtec PLUS p16/Ki-67 double-stain immunohistochemistry (IHC) on histological samples with different degrees of dysplasia, to detect cervical lesions expressing E6/E7 HPV oncogenes.

## 2. Materials and Methods

### 2.1. Cervical Tissue Specimens Selection

This study was performed in agreement with the standards of the ethics review board of “SS Annunziata” Hospital and was approved by the Ethical Committees of “G. d'Annunzio” University, in accordance with the principles outlined in the Declaration of Helsinki of 1975.

From the electronic files of Surgical Pathology Department of “SS Annunziata” Hospital of Chieti, 926 cases of biopsy-proven squamous cervical lesion, obtained from January 2010 to July 2012, were retrospectively retrieved. 

Among these casuistries, 154 cases met the following inclusion criteria:

- HPV-DNA test result by Hybrid Capture 2 (HC2), performed on liquid-based sample of exfoliated cells, collected from cervix immediately before colposcopy-directed biopsy of the lesion;
- result from mRNA testing, performed on residual cervical liquid-based cytological specimen.

Two pathologists independently reviewed haematoxylin and eosin (H&E) stained slides and reported histological diagnosis according to the World Health Organization nomenclature and criteria as follows:

 Cervical Intraepithelial Neoplasia grade 1, CIN1;
 CIN grade 2, CIN2;
 CIN grade 3, CIN3;

invasive squamous cell carcinoma (SSC). 

Only cases reaching consensus in histological diagnosis were finally included in the study (63 formalin-fixed, paraffin-embedded, FFPE).

A written informed consent was obtained from all the participants in the study, and corresponding FFPE specimens were subsequently taken. Identification codes were finally assigned to each case, in accordance with confidentiality standards.

### 2.2. Laboratory Methods

*(i) Cervical Cytology*. Cervicovaginal samples were collected from ecto-endocervix immediately before colposcopy-directed biopsy. Cervical specimens were then transferred into PreservCyt cytology medium (Cytyc Corporation, Boxborough, MA) liquid and transported to Cytopathology Departments. Cytological vials were processed using ThinPrep 2000 (Hologic, Marlborough, MA, USA). Slides were next stained with Papanicolaou procedure. 

*(ii) HPV-DNA Test*. After cytological slide preparation, an aliquot (4 mL) of each liquid-based cytological (LBC) sample, stored at RT, was removed to perform HPV-DNA testing by using the commercially available Hybrid Capture 2 system (HC2, Qiagen, Gaithersburg, MD), in accordance to manufacturer's protocol. HC2 detects oncogenic HPV types (16, 18, 31, 33, 35, 39, 45, 51, 52, 56, 58, 59, and 68). HC2 reactions were read by a luminometer, which provided a relative quantification of each individual sample in comparison to the mean of a series of positive controls containing 1 pg/mL of HPV DNA (corresponding to ~100,000 HPV-16 genomes/mL or 5,000 HPV copies per reaction). The cut-off of 1 relative light unit (RLU) was used to classify a specimen as positive or negative. RLUs value in relation to control (RLU/CO) provided an estimation of the number of HPV-DNA copies of each sample (viral load). The RLU value of each individual sample was then recorded. According to RLU/CO values, HPV-DNA positive cases were arbitrarily categorized into three groups having “low viral load” (RLU/CO from 1.0 to 50.0 RLU/CO), “intermediate viral load” (RLU/CO from 50.1 to 100.00 RLU/CO), and “high viral load” (RLU/CO > 100).

*(iii) HPV-mRNA Test*. A second aliquot (3 mL) from each residual LBC specimen was transferred into a fresh 10 mL tube for nucleic acids extraction. After centrifugation, the supernatant was removed and the sample was transferred into a tube containing 2 mL Nuclisens Lysis Buffer (BioMèrieux, France). Next, magnetized silica dioxide particles were added to the lysate to initiate the nucleic acids isolation process. Finally, nucleic acids were eluted from the solid phase in 55 *μ*L of elution buffer and stored at −20°C if not further processed immediately after extraction.

15 *μ*L of nucleic acids was used to perform mRNA testing (Nuclisens EasyQ HPV, BioMèrieux, France), in accordance with the manufacturer's instructions. 

mRNA testing is based on real-time nucleic acid sequence based amplification (NASBA) procedure, which utilizes molecular beacon probes labelled with 5-carboxyfluorescein (FAM) and Texas Red fluorochromes, at an isothermal temperature of 41°C. The test identifies full-length E6/E7 mRNA from five high-risk carcinogenic HPV types (16, 18, 31, 33, and 45). A fluorescent analyzer measured in real time the emission of the fluorescence from molecular beacon hybridized with amplified mRNA. As performance control, the human U1A mRNA from the small ribonucleoprotein-specific A protein has been used. Negative control reactions, consisting of all reagents except RNA, were performed at each run. mRNA testing was defined as positive if at least one of the five HPV genotypes detected by the test has been found [11]. 

*(iv) Chromogenic In Situ Hybridization*. Two serial sections were cut to a thickness of 4 *μ*m, one for CISH investigation and one for p16/Ki67 dual-stain IHC. The extra sections cut before and after each tissue section were stained with H&E and used to evaluate the adequacy of each FFPE for the subsequent investigations.

Bond ready-to-use DNA CISH HPV protocol (Bond ready-to-use DNA ISH HPV Probe by Leica Biosystems, Newcastle Ltd, Newcastle, UK) able to detect 5 oncogenic HPV types (types 16, 18, 31, 33, and 54) was optimized for reproducible sensitive and background free usage. Slides were then processed using the Bond-Max automated slide-staining system (Leica Biosystems, Newcastle Ltd, Newcastle, UK). Finally, CISH sections were counterstained with haematoxylin. HPV-positive controls consisted of FFPE sections containing two sets of cells: CaSki cervical cancer cell line (containing 200 to 400 copies of HPV-DNA types 16 per cell) and HeLa cervical cancer cell line (containing 10 to 50 copies of HPV-DNA types 18 per cell). Thyroid tissue has been used as negative control, since in the literature we could not find any evidence for the presence of HPV.

**Figure 1:**
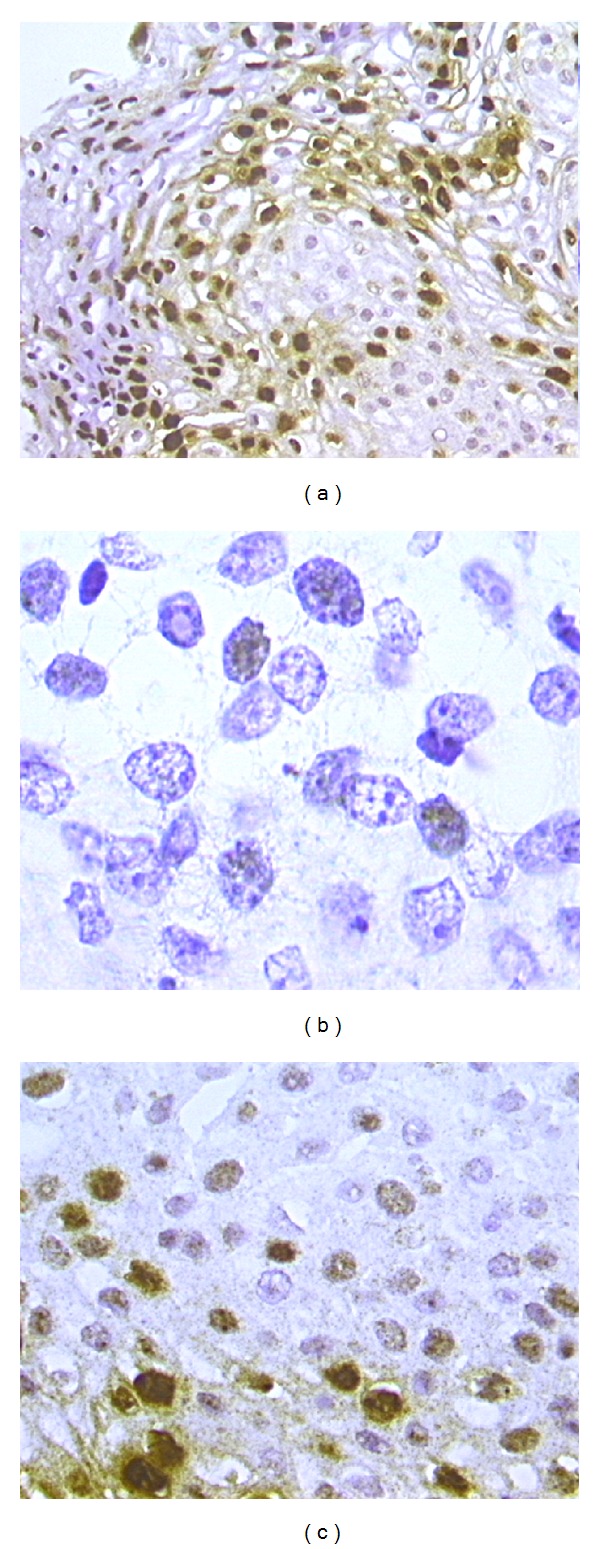
CISH positive signals. Diffuse pattern, where nuclei are completely stained ((a), 20x magnification). Punctated pattern in invasive squamous cervical cancer: distinct dot-like intranuclear signals were noted within cells infiltrating the stroma ((b), 100x magnification). Mixed patterns, where both diffuse and punctated signals are noted ((c), 40x magnification).

Two pathologists independently evaluated CISH slides. CISH signals were determined for at least 10 high power fields. Nuclear peroxidase staining was considered a positive result for HPV-DNA. Positive CISH signal patterns were classified as follows: (1) diffuse (D), when nuclei were completely stained (indicative of episomal HPV); (2) punctated, when distinct dot-like intranuclear signals were noted (indicative of integrated HPV); (3) mixed, diffuses, and punctated (D/P) when both patterns are noted. (Figures 1(a)–1(c)). 

*(v) p16/Ki67 Dual Stain and p16 Stain.* A commercial kit specifically designed for the simultaneous detection of p16 and Ki67 (CINtec PLUS Kit, Roche mtm laboratories, Heidelberg, Germany) was used, accordingly to the supplier's instructions and adapting the protocols for the use on histological samples. One section for each case was stained with p16/Ki67 dual test. A red chromogen marked Ki-67 expression within the nucleus and a brown chromogen marked cytoplasmic/nuclear p16 expression. Sample was scored as positive when the simultaneous expressions of both markers were revealed within the same cells. Cases without any double-immunoreactive cell were called negative. 

Another section for each case was prepared for the immunohistochemical evaluation of p16 alone (clone E6/H4) using CINtec Histology Kit (Roche mtm laboratories, Heidelberg, Germany). After antigen retrieval, sections were incubated with mouse monoclonal anti-p16 (Lab Vision/NeoMarkers, Fremont, CA), with EnVision+ System HRP anti-mouse (Dako, Copenhagen, Denmark). Afterwards, diaminobenzidine chromogen (Dako, Copenhagen, Denmark) was applied and counterstaining with haematoxylin was performed. p16 overexpression was visualized as a brown colour precipitate within nucleus and cytoplasm. Expression of p16 in more than 10% of epithelial cells was regarded as a positive result. 

For dual stain and p16 immunohistochemistry, positive and negative controls consisted of SCC of uterine cervix, with and without primary antibodies, respectively. All tissue slides plus controls for p16/Ki67 dual test were stained in a single session that was different from that of p16 alone. In both cases, Dako Autostainer (Dako, Copenhagen, Denmark) was used. 

Both slide sets were subjected to two pathologists, which evaluated all cases blindly to all study results. 

### 2.3. Statistical Analyses

By standard method authors calculated the prevalence of HPV-DNA, E6/E7 mRNA, p16/Ki67, and CISH positivities. Chi square or Fisher's exact test was used to assess the association between variables. Concordances between histopathological diagnosis and DNA test, mRNA test, and CISH were calculated by Kappa statistics. According to the criteria of Lands and Koch, the *K* values were divided into six scales of strength of agreement: poor (<0.00), slight (0.00–0.20), fair (0.21–0.40), moderate (0.41–0.60), substantial (0.61–0.80), or almost perfect (0.81–1.00) [12]. Chi square for trend (Cochran-Armitage test) was calculated to assess the trend of CISH results in relation with the severity of cervical disease.

Accuracy parameters (sensitivity and specificity) of each test separately as well as the comparison of accuracy parameters between tests were assessed by receiver operating characteristic analysis. Histological diagnosis was regarded as the gold standard and CIN2+ lesion was considered as the worse outcome. To do that, histological results were dichotomized into CIN2+ (including CIN2, CIN3, and SCC) and less than CIN2 (CIN2−, including CIN1). Areas under the receiver operating characteristic (ROC) curves and 95% confidence intervals (CI) were estimated to assess differences between test performances [13] and McNemar test was used for statistical significance. 

Correlation between CISH signal patterns and HPV viral load categories was evaluated by Cochran-Armitage trend test. 

Statistical analyses were performed by using SPSS software (SPSS for Windows, Inc., Chicago, IL), version 15.0. In all analyses, probability values *P* less than 0.05 were regarded as significant.

### 2.4. Results

A series of cervical FFPE from sixty-three patients (mean age 34 ± 8 years, median 33 years, range 21–63) were included in the study. Among these cases, 25 were diagnosed as CIN1, 16 as CIN2, 21 as CIN3, and 1 as SCC. 

Summary of results from histological diagnosis, HPV-DNA and mRNA tests, HPV viral load, CISH, and p16/Ki67 dual stain from each case included in the study are reported in Table 1.

*Molecular Tests*. HPV-DNA positivity was detected in 60 of the 63 (95.2%) cytological samples. Among these, 65% (*N* = 39/60) showed CIN2+ lesions in histological specimens. A positive DNA test result conferred a ≥CIN2+ odds ratio (OR) risk of 3.2 (95% CI: 0.4–26). 4.8% (*N* = 3/63) of women resulted HPV-DNA negative. Overall percent agreement between DNA testing test and histological diagnosis was 61.9% (Cohen's kappa value: 0.06, *P* < 0.05). E6/E7 mRNA positivity was detected in 71.4% (*N* = 45/63) of cytological cases; among these, 36 (80%) were CIN2+. Within the 18 mRNA negative cases, 16 (88.9%) were confirmed as CIN2−. mRNA test results were associated to CIN2+ diagnosis with a OR = 32 (95% CI: 7–144). Overall percent agreement between mRNA testing and histological diagnosis was 82.5% (Cohen's kappa value: 0.62, *P* < 0.0001).

Diagnostic performances of both DNA and mRNA tests are represented in Table 2. mRNA test improved specificity of DNA testing. Difference was statistically significant (McNemar test, *P* < 0.01).

**Figure 2:**
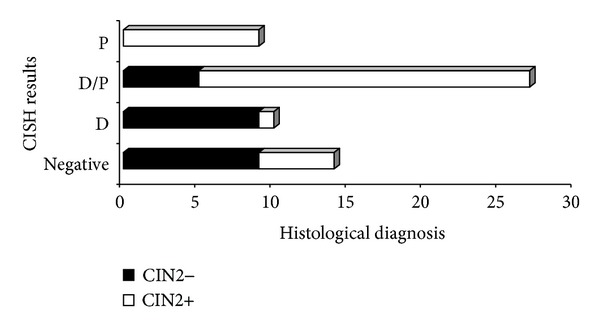
Correlation between CISH results and histological diagnosis (P < 0.0001). P: punctate pattern; D/P: diffuse and punctated (mixed) pattern; D: diffuse pattern. CIN2+: Cervical intraepithelial neoplasia grade 2 or greater (including CIN2, CIN3, and invasive squamous cell carcinoma); CIN2−: less than Cervical Intraepithelial Neoplasia grade 2 (including CIN1 and negative for dysplasia.

*CISH Results*. CISH showed a clear signal in 95.2% (*N* = 60/63) of the specimens. Invalid result has been found in 4.8% (*N* = 3/63) of the cases, due to unclear and weak signal. The rate of positive results was 73% (*N* = 46/63). Among these, 30.4% (*N* = 14) were CIN1, 30.4% (*N* = 14) were CIN2, and 37% (*N* = 17) were CIN3. The unique case of SCC showed CISH positivity. Negativity has been found in 22.2% (*N* = 14/63) of the cases.  Table 3 shows details of the distribution of CISH signal patterns and their correlation with histological diagnosis. As expected, CISH showed a clear punctated signal pattern in both HPV positive cell lines, whereas no signal was detected in thyroidal tissue. Nonspecific background binding has never been seen among the 60 cases which were considered as valid cases. Notably, about two-thirds of diffuse pattern were associated with CIN1, while the unique case of SCC displayed a punctated pattern. Differences were statistically significant (*P* < 0.01). Dichotomizing histological diagnosis and considering only CISH-positive results, diffuse pattern has been found in 64.3% (*N* = 9/14) of CIN2− and 3.1% (*N* = 1/32) of CIN2+. All cases of punctated pattern have been found in CIN2+, as well as 68.8% of mixed patterns. The proportion of punctated pattern increased with the severity of cervical lesion (Cochran-Armitage test for trend *P* < 0.0001) (Figure 2).

Sensitivity and specificity of CISH analysis were 86.5% (95% CI: 71.4–94.4) and 39.1% (95% CI: 22.2–59.3), respectively. A positive CISH result conferred a ≥CIN2+ risk (OR) of 4.11 (95% CI: 2–13.9).

**Figure 3:**
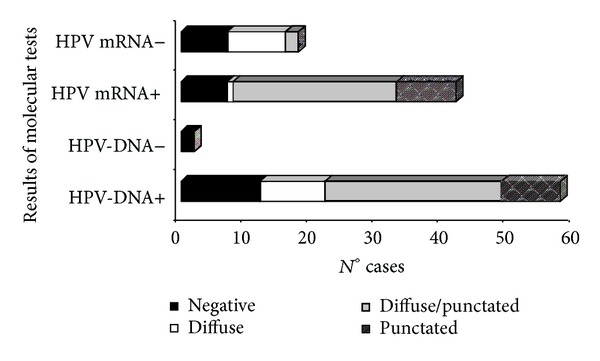
Correlation between CISH signal and results from molecular tests (*k* = 0.20, *P* < 0.05).

CISH results were assessed against HPV-DNA test (Figure 3). All CISH-positive cases also resulted HPV-DNA positive. Among HPV-DNA positive patients, 76.7% (*N* = 46/60) were CISH positive. Within CISH-negative cases, 85.7% (*N* = 12/14) were HPV-DNA positive, while 14.3% (*N* = 2/14) were HPV-DNA negative (*P* = .001). Overall percent agreement between CISH and DNA test was 80% (*k* = 0.20, *P* < 0.05).

CISH results were also assessed against HPV E6/E7 mRNA expression. Among mRNA+ cases, 77.8% (*N* = 35/45) were CISH positive. Of those, 2.9% (*N* = 1/35) showed a diffuse pattern, 71.4% (*N* = 25/35) a mixed pattern, and 25.7% (*N* = 9/35) a punctated pattern. Among the 11 mRNA/CISH+ cases, only 2 cases (18%) demonstrated a punctated pattern (*P* < 0.0001). Overall percent agreement between CISH and mRNA test was 70% (*k* = 0.24, *P* < 0.05).

**Figure 4:**
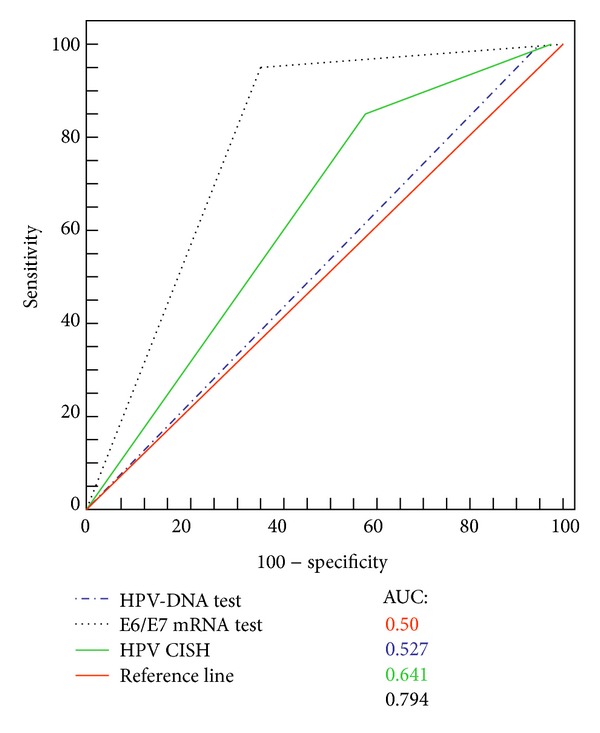
Receiving operating characteristic curves (ROC), comparing CISH, HPV-DNA test, and E6/E7 mRNA diagnostic performances. The red line indicates a reference threshold with area under the ROC curve of 0.5.

Since HPV-DNA test is currently considered the most reliable method to detect papillomavirus infection in both cytological and histological samples, the performances of CISH and mRNA test were compared to HPV-DNA test performance. DNA testing achieved an area under the curves (AUC) of 0.53 (95% CI, 0.4–0.65) CISH and of 0.64 (95% CI, 0.5–0.75) and mRNA testing of 0.79 (95% CI, 0.67–0.89) (Figure 4). Difference between HPV-DNA test and mRNA test was statistically significant (*P* < 0.0001), while difference between RNA testing and CISH did not reach significance (*P* = 0.06).

*CISH Results and HPV Viral Load.* Among cytological samples testing HPV-DNA positive, the mean of viral loads was 502.9 ± 620.5 RLU/CO, the median being 155.79 RLU/CO (range 1.45–2663.29 RLU/CO). 

**Figure 5:**
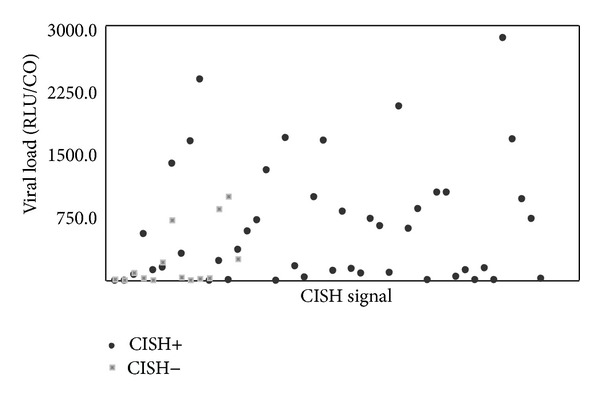
Correlation between CISH signal and HPV viral load, as detected by HC2 test (*P* = 0.01). RLU/CO value provided an estimation of the number of HPV-DNA copies of each sample. RLU/CO: ratio between relative light units and control.

Considering the categories of viral load values as described in Section 2, 31.7% (*N* = 19/60) of the cases showed low viral load, 6.7% (*N* = 4/60) intermediate load, and 61.6% (*N* = 37/60) high viral load. The rate of CISH positivity has been found to be lower in cases with low viral load level (58%) than in those with intermediate (75%) and high (86.6%) load levels (Cochran-Armitage trend test, *P* = 0.01) (Figure 5).

Correlation between CISH punctate signal pattern and viral load categories showed that the rate of this pattern was higher in specimens with low viral loads than in those having intermediate or high loads (Fisher exact test, *P* = 0.05).

*p16 and p16/Ki67 Dual Stain Analysis*. Both p16 immunohistochemistry and p16/Ki67 analysis were performed on the entire FFPE series. 

A positive p16 result was defined as a diffuse moderate-to-strong cytoplasmic and nuclear staining. There was no difference in the intensity of staining between the different epithelial layers. Brown staining of normal metaplastic or endocervical cells was considered as negative p16 test. 

When the diagnosis of cervical lesion was categorized into four, that is, CIN1, CIN2, CIN3, and SCC, a complete concordance for all the two observers was obtained in 32 cases (51%), including 8 CIN1, 11 CIN2, 12 CIN3, and 1 SCC (*k* = 0.06). The lower agreement was observed for CIN1 diagnosis, the higher for SCC (*P* = 0.08) (Table 4). Sensitivity and specificity of p16 IHC were 96.4 (95% CI: 85.1–100) and 100% (95% CI: 83.9–100), respectively.

**Figure 6:**
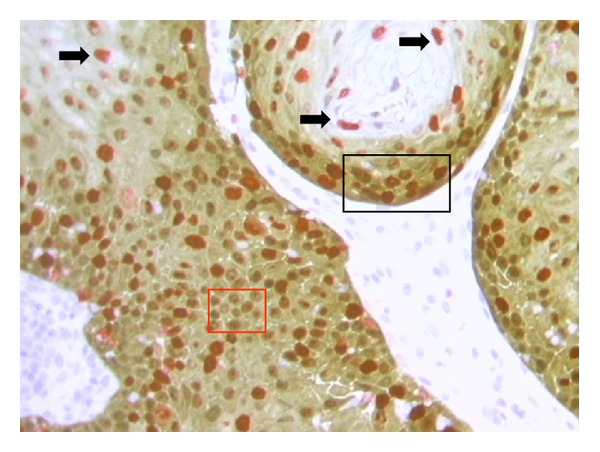
p16/Ki67 dual stain (40x magnification). Red square: brown chromogen marked cytoplasmic/nuclear p16 expression. Black arrows: red chromogen marked Ki-67 expression within nuclei. Black square: simultaneous expressions of both markers were revealed within the same cells.

Considering p16/Ki67 dual stain immunohistochemistry (Figure 6), all 63 histological samples gave interpretable results. p16 expression was observed in 48 of 63 cases (76.2%). Ki67 expression has been found in all histological specimens. Particularly, 13/25 CIN1 cases (52%) showed weak Ki67 expression in the basal layer of cervical epithelium. The remaining 12 CIN1 cases showed strong nuclear Ki67 expression in the lower part of the epithelium (one-third), associated with cytoplasmic expression of p16 within the same cells. As the CIN grade was higher, stronger Ki67 expression was observed, particularly in 87.5% of CIN2 (*N* = 14/16) cases (within two-third of cervical epithelium) and in 100% of CIN3 cases (within the three-third of the epithelium). Expression level of p16 positively correlated with that of Ki67 (*P* < 0.01). In the unique case of SCC, strong dual-stain positivity has been also shown by neoplastic cells infiltrating the stroma.

The use of p16/Ki67 IHC significantly improved consensus among pathologists, which reached 100% (*k* = 1). Sensitivity and specificity of dual stain were 100% (95% CI: 88.8–100) and 84% (95% CI: 64.6–94.1), respectively.

Since in cervical tissue p16 is considered a surrogate biomarker of HPV-E7 expression, we correlated both p16 and p16/Ki67 staining results with HPV-E6/E7 status, as determined by mRNA test (Table 5). p16 expression was observed in 77.8% (*N* = 35/45) of mRNA-positive cases. Among mRNA-negative cases, p16 showed no immunoreactivity in 88.9% (*N* = 16/18) of patients. Concordance between p16 and E6/E7 mRNA test was 81% (*k* = 0.59).

Dual stain positivity has been found in 88.9% (*N* = 40/45) of mRNA-positive patients, negativity being detected in 88.8% (*N* = 16/18) of mRNA-negative cases. Concordance between p16/Ki67 dual stain and mRNA test was 89% (*k* = 0.74). 

Concordance between dual stain and CISH (punctated and mixed pattern) was 83.3% (*k* = 0.64). 

## 3. Discussion

Although HPV-DNA and E6/E7 mRNA tests still remain the current standards for the confirmation of HPV infections in cytological specimens, no consensus exists about technology that should be used for the detection of Papillomavirus in formalin-fixed paraffin-embedded samples [14]. This fact presents the clinicians with the dilemma of selecting the more suitable method. Molecular techniques (such as PCR) certainly represent the gold standard method, reaching a sensitivity of 1 DNA copy/cell [14]. However, DNA extraction requires trained laboratory personnel and is still highly time-consuming and labour intensive for routine application. In addition, to detect HPV-DNA, a wide range of consensus primers, such as MY09/11, PGMY09/11, GP5+/6+, and SPF, are available [15]. Amplification with each of these primers provides amplification products of different sizes, thus providing different levels of sensitivity in viral detection. Particularly on FFPE material, because of the damaged and fragmented DNA, it is possible that the use of these primers could reach a high rate of false negative results [14]. It has been already shown that the maximum accuracy of PCR is obtained using fresh frozen tissues [16]. 

All consensus PCR primers for the detection of HPV-DNA would target L1 region. This region is deleted when HPV-DNA is integrated into the host cell genome [17]. So, when HPV integration would occur, PCR should probably give false negative results. 

Finally, due to its high sensitivity, PCR would detect HPV infection without any correlation with the prognosis of cervical lesion. 

ISH is certainly less sensitive than PCR [18], but the visualization of HPV-DNA signals within nuclei of cervical lesions could offer both detection and localization of HPV-DNA without damage of morphology. In addition, ISH helps to distinguish between episomal HPV from integrated one, the last being the necessary condition for neoplastic progression [19]. However, the low analytic sensitivity of ISH, ranging from 10 to 50 copy/cell, would be a weakness in case of high-grade cervical lesions in which, due to the frequent integration status of HPV, DNA copy number is usually less than 50 copy/cell. [7, 8]. Then, the choice of ISH technique would be extremely important. Non-tyramide-based methods showed too low sensitivity rate [20, 21]. On the other hand, the higher sensitivity of tyramide-based ISH could lead to interpretation bias, especially due to non-specific staining [22]. Hence, our aim is to analyse the performances of an optimized chromogenic ISH tyramide-based biotin-free assay. 

In our FFPE series, sensitivity of CISH was about 87%, higher if compared to series using non-tyramide-based methods [19, 22, 23]. CISH positive cases were characterized by a clear background. The rate on invalid results was very low (4.8%) and due to scant FFPE specimens. CISH positivity always matched with HPV-DNA positivity. 20% of HPV-DNA positive cases demonstrated negativity at CISH analysis. The latter data may probably be due to the limited number of oncogenic genotypes detected by CISH probe (HPV 16, 18, 31, 33, and 54), in comparison to those detected by HC2. Anyhow, HPV types identified by CISH would represent five of the six most oncogenic genotypes, the sixth being HPV-45 [1, 24–26]. 

It is now well known that HPV integration is common in CIN2+ lesions while is uncommon or absent in CIN1 [27]. Studies on cervical carcinomas and SCCs cell lines demonstrated that oncogenic E6/E7 oncogenes are frequently overexpressed during HPV integration [27]. In our study, 100% of cases with punctate signal matched with CIN2+, while 94.7% of CIN2+ showed E6/E7 oncogenic expression (E6/E7 mRNA positivity). Percent agreement between CISH and mRNA test was high. Thus, we may conclude that CISH punctate signal confirmed as a sign of viral integration [18]. The only two CISH+/mRNA cases were probably due to HPV genotype 54, detected by CISH but not detected by mRNA test.

In our series, when present, diffuse signal has been detected within cells of the mid/superficial layers. This pattern was mainly associated with CIN2−/mRNA negative cases and confirmed as a marker of productive HPV infections. Diffuse and punctate signals within the same lesion have also been found. This mixed pattern was associated with CIN2+ in 81.5% of the cases. This fact would be due to the polyclonal nature of cervical intraepithelial lesions. The unique case of infiltrating SCC showed punctate signal only, confirming the monoclonality of invasive neoplasia. 

Although our cohort encompassed a limited number of cases, our preliminary results underline the usefulness of the tyramide-based CISH protocol which we used. This technology does not suffer of nonspecific background, simultaneously allowing the detection of HPV genome within morphological context. In addition, the use of a chromogen in alternative to fluorescence revealed to be more convenient for routine purpose, given the wide availability of light microscopy in pathology settings. Finally, CISH protocol could prove helpful also during the followup of patients with cervical lesions, as a feasible alternative to HPV-DNA and E6/E7 mRNA tests on FFPE specimens. 

Recent researches on cervical cancer widely analysed biomarkers resulting associated with the various stages of HPV infection [3]. One of these strongly related to transforming HPV infection would be p16. Overexpression of p16 seems to increase with increasing degree of cervical lesion [28, 29]. A meta-analysis on p16 immunostaining on cytological and histological cervical specimens estimated that 2% of normal tissues and 38% of CIN1 showed diffuse staining, compared with 68% of CIN2 and 82% of CIN3 [30]. p16 immunostaining demonstrated to be cheap and easy to perform in pathology laboratories. The semiquantitative scoring system described by Klaes et al. [31], is actually the most widely used approach for the evaluation of this marker on histological specimens. However, estimation of results is often based on colorimetric and morphological criteria which are often subjective. This lack of standardization would make the use of this biomarker somehow difficult [30]. The assessment of p16 staining can be also hampered by false positive results [32, 33]. Endometrial, metaplastic, and endocervical cells, as well as tubo-endometrioid metaplasia would stain p16-positive [34], since a non-HPV dependent p16 expression pathway may also exist [4, 35]. For all the above mentioned reasons, there would be considerable reluctance among histopathologists to incorporate p16 IHC into routine gynae-pathology. Specifically, in our series the evaluation of p16 immunoreactivity generated a great variability in the interpretation and reached a low agreement level (51%).

Nowadays, there is a considerable interest in the evaluation of the combination p16/Ki67, which would allow to differentiate dysplastic cells from nondysplastic ones, and meaningless HPV infection from transforming ones. In the present study, we performed p16/Ki67 dual stain immunohistochemistry on FFPE series of specimens encompassing all grades of morphological abnormalities. In our experience, this genotype-independent method has proved to be feasible and highly efficient in producing valid results. Even though in a limited series, dual stain results were always unequivocal. Moreover, inter-observer agreement was highest (100%), since only cells simultaneously showing p16/Ki67 expression have been considered as positive, irrespective of morphology. Finally, in our series dual stain improved specificity of p16 alone. 

In this setting, 98% of CIN2+ stained mRNA positive, while 100% stained p16/Ki67-positive. The only invasive cancer showed dual stain and E6/E7 mRNA positivity. It seems likely that dual stain positive/mRNA positive CIN2+ could represent cervical lesions at higher risk of progression toward invasive cancer [36]. This fact could not be determined in the present setting, since all CIN2+ lesions were surgically removed [37].

## 4. Conclusion

HPV are recognized as a necessary cause of CIN, but only a minority of HPV infections even results in cervical lesions. Although the majority of infections may be cleared by immune system, integration of HPV sequence into the host genome may induce CIN progression. The detection of HPV genome within cervical lesions and the assessment of its physical status are then crucial in prognostic terms.

To the authors' knowledge, this is the first study evaluating the novel HPV tyramide-based CISH technology and the innovative CINtec PLUS p16/Ki-67 double stain immunohistochemistry on histological tissues, as well as the first investigation comparing both methods to molecular tests actually considered as the gold standards for HPV detection.

Molecular assays may be expensive and require a high level of expertise, which are often difficult to reach in routinely laboratory. Although larger studies are needed, our data demonstrate the usefulness of CISH and p167Ki67 immunostaining in surgical pathology settings. 

In particular, CISH could be a feasible method to localize HPV genome on paraffin-embedded specimens. This technology would help to distinguish episomal from integrated HPV, thus allowing conclusions regarding the prognosis of the lesion. Likewise, the genotype-independent p16/Ki67 dual staining approach, which demonstrated greater efficacy than p16 alone, would confer a higher level of standardization to the diagnostic procedure.

Finally, due to their strong correlation with tests which are currently considered the standards for HPV detection in cytological specimens, both CISH and dual stain technologies would be considered a viable potential alternative to molecular assays in the evaluation of the biology of cervical lesions. 

Nevertheless, these preliminary data need to be confirmed in a larger clinical cohort.

## Figures and Tables

**Table 1:** Summary of results from histological diagnosis, HPV-DNA and mRNA tests, HPV viral load, CISH, and p16/Ki67 dual stain.

Case	Histological diagnosis	HR HPV-DNA test result	HC2 viral load (RLU/CO)*	E6/E7 mRNA test result	Type specific mRNA test result**	HPV CISH^†^ signal	P16/Ki67 Dual stain result
1	CIN1	Positive	63.5	Negative	—	*P *	Negative
2	CIN1	Positive	516.61	Negative	—	*D *	Negative
3	*CIN3 *	Positive	*15.79 *	Negative	—	*Negative *	Positive
4	CIN2	Positive	146.77	Positive	16	*D/P *	Positive
5	CIN2	Positive	1283.97	Negative	—	*D/P *	Positive
6	CIN1	Positive	298.55	Negative	—	*D *	Negative
7	CIN3	Positive	*1529.35 *	Positive	16, 31	*D/P *	Positive
8	CIN3	Positive	2207.8	Positive	31	*D/P *	Positive
9	CIN1	Positive	2.06	Negative	—	*D *	Negative
10	*CIN2 *	Positive	*216.93 *	Positive	31	*D/P *	Positive
11	CIN1	Positive	6.7	Negative	—	*Negative *	Negative
12	CIN2	Positive	84.32	Positive	31	*Negative *	Positive
13	CIN3	Positive	10.56	Positive	16	*D/P *	Positive
14	CIN3	Positive	338.02	Positive	16	*D/P *	Positive
15	CIN1	Positive	1211.84	Positive	33	*D/P *	Positive
16	CIN3	Positive	3.3	Positive	16	*P *	Positive
17	CIN1	Positive	155.79	Positive	16	*D *	Negative
18	CIN1	Positive	28.81	Negative	—	*Negative *	Positive
19	CIN2	Positive	34.82	Negative	—	*D *	Positive
20	CIN3	Positive	913.36	Negative	—	*D *	Positive
21	CIN2	Positive	1536.02	Positive	16	*P *	Negative
22	CIN2	Positive	107.22	Positive	18, 45	*D/P *	Positive
23	CIN1	Positive	75.86	Negative	—	*D/P *	Negative
24	CIN2	Positive	675.75	Positive	16	*D/P *	Positive
25	CIN2	Positive	596.84	Positive	16	*D/P *	Positive
26	CIN3	Positive	1914.17	Positive	16	*D/P *	Positive
27	CIN2	Positive	570.26	Positive	16, 45	*D/P *	Positive
28	CIN1	Positive	783.56	Negative	—	*D *	Negative
29	CIN1	Negative	—	Negative	—	*Negative *	Negative
30	CIN3	Positive	3.93	Positive	16	*P *	Positive
31	CIN3	Positive	968.56	Positive	31	*D/P *	Positive
32	CIN1	Positive	926.05	Positive	31	*Invalid *	Negative
33	CIN2	Negative	—	Positive	31	*Invalid *	Positive
34	CIN1	Positive	45	Negative	—	*D *	Negative
35	CIN3	Positive	117	Positive	16	*D/P *	Positive
36	CIN1	Positive	237.48	Positive	16	*Invalid *	Negative
37	CIN1	Positive	5.31	Positive	18, 31	*Negative *	Negative
38	CIN1	Positive	204.42	Positive	45	*Negative *	Negative
39	CIN3	Positive	663.26	Positive	33	*Negative *	Positive
40	CIN3	Positive	38.61	Positive	16	*Negative *	Positive
41	CIN1	Positive	10.11	Positive	33	*D/P *	Positive
42	CIN1	Positive	137.88	Negative	—	*D *	Negative
43	CIN3	Positive	6.21	Positive	16	*Negative *	Positive
44	CIN2	Positive	2663.26	Positive	31	*D/P *	Positive
45	SCC	Positive	1549.74	Positive	33	*D/P *	Positive
46	CIN2	Positive	894.17	Positive	16	*P *	Positive
47	CIN1	Positive	20.47	Negative	—	*Negative *	Negative
48	CIN1	Positive	26.23	Negative	—	*Negative *	Negative
49	CIN1	Positive	787.16	Positive	31	*Negative *	Negative
50	CIN1	Positive	676.46	Positive	16	*D/P *	Negative
51	CIN3	Positive	2.36	Positive	16	*P *	Positive
52	CIN3	Positive	1.45	Positive	18	*P *	Positive
53	CIN3	Positive	111.95	Positive	16, 18	*D/P *	Positive
54	CIN3	Positive	544.41	Positive	16	*D/P *	Positive
55	CIN2	Positive	663.21	Positive	16	*D/P *	Positive
56	CIN1	Negative	—	Negative	—	*Negative *	Negative
57	CIN2	Positive	1569.56	Positive	18	*D/P *	Positive
58	CIN3	Positive	758.66	Positive	16	*D/P *	Positive
59	CIN2	Positive	130.13	Positive	16	*D/P *	Positive
60	CIN3	Positive	87.01	Positive	16	*P *	Positive
61	CIN3	Positive	968.56	Positive	31	*D/P *	Positive
62	CIN2	Positive	6.21	Positive	16	*P *	Positive
63	CIN3	Positive	24.01	Positive	16	*P *	Positive

*Relative light unit in relation to control (RLU/CO).

**HPV genotype(s) detected by Nuclisens EasyQ HPV mRNA test.

^†^D: diffuse; P: punctated; D/P: mixed diffuse/punctated.

**Table 2:** Diagnostic performances of HPV-DNA test (HC2) and E6/E7 mRNA test.

Molecular testing	Diagnostic performances (95% CI*)	
	Sensitivity	Specificity
HPV-DNA test	97.4% (85.1–100)	8% (1.2–26)
HPV-mRNA test	90.7% (81.6–99.4)	64% (44.4–79.7)

*Confidence intervals (CI).

**Table 3:** Association between CISH signal patterns and grading of cervical lesions (*P* < 0.01).

CISH result	Number of cases (%)				Total
	CIN1	CIN2	CIN3	SCC	
Invalid	2 (8)	1 (6.3)	0	0	**3 (4.8)**
Negative	9 (36)	1 (6.3)	4 (19)	0	**14 (22.2)**
Diffuse	9 (36)	1 (6.3)	0	0	**10 (15.9)**
Diffuse-punctated	5 (20)	11 (68.7)	11 (52.4)	0	**27 (42.8)**
Punctated	0	2 (12.4)	6 (28.6)	1 (100)	**9 (14.3)**
Total	**25 (39.7)**	**16 (25.4)**	**21 (33.3)**	**1 (1.6)**	**63**

**Table 4:** p16 immunostaining: interobserver agreement within histological categories of cervical lesions.

p16 interobservers agreement	Histological diagnosis				
	CIN1 (%)	CIN2 (%)	CIN3 (%)	SCC (%)	Total
Positive	8 (32)	11 (68.8)	12 (57.1)	1 (100)	**32 (50.8)**
Negative	17 (68)	5 (31.2)	9 (42.9)	0	**31 (49.2)**
Total	**25 (39.7)**	**16 (25.4)**	**21 (33.3)**	**1 (1.6)**	**63**

**Table 5:** Correlation between p16 and p16/Ki67 immunohistochemistry and E6/E7 mRNA test.

Immunohistochemistry	E6/E7 mRNA test		
	Positive (%)	Negative (%)	Total
p16 positive	35 (77.8)	2 (11.1)	**37 (58.7)**
p16 negative	10 (22.2)	16 (88.9)	**26 (41.3)**
Total	**45**	**18**	**63**
p16/Ki67 dual stain positive	40 (88.9)	2 (11.1)	**42 (66.7)**
p16/Ki67 dual stain negative	5 (11.1)	16 (88.8)	**21 (33.3)**
Total	**45**	**18**	**63**
